# THE PARTNERSHIP BETWEEN THE CENTER FOR SCIENTIFIC REVIEW AND THE NIH INSTITUTE

**DOI:** 10.1093/geroni/igac059.113

**Published:** 2022-12-20

**Authors:** Dana Plude

**Affiliations:** National Institute on Aging/National Institutes of Health, Bethesda, Maryland, United States

## Abstract

The playing field for NIH funding is highly competitive, and having a great idea is only the start of the process. There are many funding mechanisms for early career investigators, trainees, and research institutions. Understanding about different application types, who to talk to about your application at different stages of development, finding the right review panel, and learning about the policies and procedures pertaining to review are important first steps in preparing an application. In recent years, the Center for Scientific Review has undergone changes and updated its structure, policies and practices to stay aligned with its mission. In this presentation, attendees will learn about the roles played by CSR and the NIH Institute as applications move from review to funding.

